# Long Noncoding RNA TUG1 Inhibits Tumor Progression through Regulating Siglec-15-Related Anti-Immune Activity in Hepatocellular Carcinoma

**DOI:** 10.1155/2022/9557859

**Published:** 2022-02-21

**Authors:** Yanyi Ren, Jiayi Lyu, Yaoguang Guo, Yuan Yao, Lin Hu

**Affiliations:** ^1^Department of Gastroenterology, Hospital of Chengdu University of Traditional Chinese Medicine, Chengdu, China; ^2^Department of Clinical Laboratory, Hospital of Chengdu University of Traditional Chinese Medicine, Chengdu, China; ^3^Department of Acupuncture and Moxibustion, Hospital of Chengdu University of Traditional Chinese Medicine, Chengdu, China; ^4^Department of Neurosurgery, Hospital of Chengdu University of Traditional Chinese Medicine, Chengdu, China; ^5^Department of Medical Imaging, Hospital of Chengdu University of Traditional Chinese Medicine, Chengdu, China

## Abstract

**Background:**

Hepatocellular carcinoma (HCC) is the second leading cause of cancer-related death, and its biology remains poorly understood, especially in regards to the immunosuppression induced by immune checkpoints, such as Siglec-15. Most cancer treatments composed of immune checkpoint inhibitors and oncogene-targeted drugs display a better therapeutic effect in the clinic, including tumor progression inhibition and immunosuppression breaks. However, two or more drugs will result in a greater possibility of adverse effects. Thus, a double-function target is necessary for developing antitumor drugs, such as RNAi therapy.

**Methods:**

The expression of TUG1, Siglec-15, and miRNAs was evaluated by qPCR, and protein expression was analyzed by western blotting. The immune responses were evaluated by a Jurkat-reporter gene assay, a T cell-induced cytotoxicity assay, and IFN-*γ*/IL-2 release. The interactions among TUG1, Siglec-15, and miRNAs were verified by dual-luciferase reporter, RNA immunoprecipitation, and RNA pull-down assays. CCK-8 and Transwell assays were used to determine tumor cell proliferation, migration, and invasion.

**Results:**

In HCC patients and cells, increased TUG1 levels were observed, positively regulating Siglec-15 expression. TUG1-induced Siglec-15 upregulation resulted in the suppression of the immune response of HCC cells. hsa-miR-582-5p directly targeted TUG1 and Siglec-15 mRNA, and ihsa-miR-582-5p knockout prevented the regulation of Siglec-15 induced by THU1. Changes in hsa-miR-582-5p expression negatively regulated Siglec-15 levels and immunosuppression but had no influence on TUG1 levels. siRNA knockdown of TUG1 effectively led to tumor progression inhibition and immune response improvement in HCC cells both in vitro and in vivo.

**Conclusion:**

TUG1 increases the Siglec-15 level in HCC cells as a sponge to hsa-miR-582-5p, resulting in enhanced immunosuppression. TUG1 knockdown induced by siRNA not only reduces immunosuppression but also suppresses tumor progression both in vitro and in vivo. These novel findings may provide a potential and appropriate target for RNAi therapy to develop drugs with dual antitumor activity.

## 1. Introduction

Hepatocellular carcinoma (HCC) is the second leading cause of cancer-related death and the sixth most prevalent cancer worldwide with increasing incidence in recent decades [1]. Although developments in surgical techniques have enhanced the prognosis of patients with HCC, the overall survival is still poor with a median survival of 9 months and a 3-year survival rate of 12.7% [2]. Thus, developing more reliable and novel strategies is required to improve treatment efficacy.

Recently, a new therapeutic strategy, RNA interference (RNAi), has received much attention. RNAi is a ubiquitous posttranscriptional gene silencing (PTGS) mechanism found in metazoan cells. mRNAs encoding proteins or other noncoding RNAs are inhibited by small duplex RNAs, such as short interfering RNAs (siRNAs) and microRNAs (miRNAs). Most RNAi drugs in preclinical or clinical trials use siRNA due to its high specificity compared to miRNAs [3]. Despite the challenge of RNAi delivery, liver-targeted RNAi delivery is relatively established using GalNAc-siRNA conjugates with several applications in clinical trials [4]. Thus, it is possible to treat HCC using the RNAi technology if there are appropriate target genes.

Recently, sialic acid-binding immunoglobulin-like lectin 15 (Siglec-15), one of the Siglec gene family members with a characteristic sialic acid-binding immunoglobulin-type lectin structure [5], has been demonstrated to be an immune checkpoint and called a next-generation immunooncology agent [6, 7]. In contrast, the regulation of Siglec-15 in HCC is limited. Long noncoding RNAs (lncRNAs) are RNAs without protein-coding ability and are longer than nucleotides [8]. Taurine-upregulated gene 1 (TUG1) is a lncRNA that was first identified in taurine-treated mouse retinal cells [9]. Many reports have demonstrated that TUG1 knockdown inhibits cancer progression and suppresses tumor growth in many cancers, including HCC [10–13]. However, the regulatory role of TUG1 in immune checkpoints, such as PD-L1/PD1, CTAL-4, and Siglec-15, is still unknown, especially in HCC.

Here, we explored the function of TUG1 in HCC to regulate Siglec-15 as a sponge to target hsa-miR-582-5p, thereby downregulating Siglec-15 expression. In addition, we designed and screened siRNAs targeting the conserved region of TUG1 as RNAi drug candidates, which inhibited tumor growth and progression as well as improved T cell-induced antitumor immune activity both in vitro and in vivo. Thus, TUG1 may be an appropriate target for RNAi therapy in HCC.

## 2. Methods and Materials

### 2.1. Bioinformatics Strategy for Identification of lncRNAs

Briefly, we first identified the lncRNAs with abnormal expression in HCC according to the Lnc2Cancer database (http://biobigdata.hrbmu.edu.cn/lnc2cancer/) and found 513 lncRNAs, including 340 upregulated lncRNAs. We then analyzed the correlation between Siglec-15 and the 340 lncRNAs using the StarBase database (http://starbase.sysu.edu.cn/starbase2/index.php). Only 45 lncRNAs showed correlation, and 25 of these showed a positive correlation with Siglec-15. The reason for focusing on lncRNAs upregulated in HCC and positively correlated with Siglec-15 was to develop siRNA drugs inhibiting Siglec-15 through downregulating lncRNAs, which may have other synergistic effects. We also used TCGA to confirm the remaining 25 lncRNAs, excluding 8 lncRNAs. Thus, we hypothesized that these remaining lncRNAs play a significant role in HCC progression and development, and we also hypothesized that Siglec-15 regulation influences the survival of HCC patients.

### 2.2. Cell Culture

The LO-2 human normal hepatic cell line and the QGY-7701 and HUH7 HCC cell lines were obtained from ATCC. The HepG2, Bel-7404, SK-Hep-1, and Hep3B HCC cell lines were obtained from the National Collection of Authenticated Cell Cultures (NCAC) in China. Primary human hepatocytes (HH, mixed donor) were obtained from Lonza (HUCS10P). Cell lines were cultured with Eagle's minimum essential medium (EMEM, Gibco) supplemented with 10% fetal bovine serum (FBS, Gibco). HH cells were cultured in HCM™ Hepatocyte Culture Medium BulletKit™ (CC-3198, Lonza). All cells were incubated at 37°C and 5% CO_2_. hsa-miR-582-5p knockout (miRKO) cell lines were generated by GenePharma with the CRISPR/Cas9 system.

### 2.3. Transfection and Transduction

The siRNAs, miRNAs, miRNA inhibitors and negative controls were synthesized by Sangon Biotech (China). Plasmids for transient overexpression of TUG1 in the pCDA3.1 backbone and lentiviruses containing TUG1-IRES-EGFP, TUG1, and sh-TUG1 were generated by GenePharma. The integration titer of lentivirus was 1 × 10^8^ TU/ml (quantified by GenePharma). Transfection was performed when cells reached 50-60% confluence using Lipofectamine 3000 reagents (Thermo Fisher Scientific) according to the manufacturer's instructions. Lentiviral transduction was performed when cells reached 70-80% confluence with a MOI of 20. Twenty-four hours after transduction, the medium was removed and replaced with fresh medium containing 2 *μ*g/ml puromycin (Sigma), and cells were cultured for two weeks. Stable cell lines were maintained by culturing with medium containing 2 *μ*g/ml puromycin.

### 2.4. Quantitative Polymerase Chain Reaction (qPCR)

miRNA was extracted by TRIzol reagent (Sigma), and reverse transcription was performed by an One-Step miRNA cDNA Synthesis Kit (Takara). mRNA was also obtained using TRIzol reagent and transcribed by a PrimeScript RT reagent kit (Takara). mRNA expression was measured by the TB Green® Fast qPCR Mix (Takara). The measurement of miRNAs was performed using stem–loop qRT–PCR (TaqMan method) (Applied Biosystems). The primers and probes for miRNA qPCR were obtained from TaqMan® MicroRNA Assays (Applied Biosystems). The following qPCR primers were used: Siglec-15-F, 5′-TTCTCCCGACAGGCTCATTT-3′; Siglec-15-R, 5′-TGTGCACCTCTGTGTTGAGC-3′; TUG1-F, 5′-ACGACTGAGCAAGCACTACC-3′; TUG1-R, 5′-CTCAGCAATCAGGAGGCACA-3′; GAPDH-F, AGGTCGGAGTCAACGGATTT; and GAPDH-R, TGGAATTTGCCATGGGTGGA.

### 2.5. Western Blot Analysis

Cells were lysed with RIPA buffer (Sangon), and proteins were quantified using a BSA Kit (Beyotime, Shanghai, China). Tumor proteins were extracted by a One-Step Animal Tissue Active Protein Extraction kit (Sangon). Protein (20 *μ*g) was loaded and separated by 12% sodium dodecyl sulfate–polyacrylamide gel electrophoresis (SDS–PAGE) (Fdbio Science, Hangzhou, China) at 80 V for 30 min and then 120 V for 60 min followed by transfer to polyvinylidene fluoride (PVDF) membranes (Millipore) at 300 mA for 90 min. After blocking with 5% fat-free milk for 1 h, the membranes were incubated with primary antibodies overnight at 4°C. The following primary antibodies were used: anti-Siglec-15 (1 : 1000, Santa Cruz), anti-GAPDH (1 : 5000, Sino Biological), anti-AGO2 (1 : 20, Abcam), anti-C-caspase3 (1 : 1000, Abcam), anti-caspase3 (1 : 1000, Abcam), and anti-Bcl-2 (1 : 1000, Abcam). After incubation with HRP-conjugated antibodies (1 : 5000, Sino Biological) for 2 h at room temperature, an ECL kit (Fdbio Science) was used for visualization of protein bands.

### 2.6. Jurkat-Based Reporter Gene Assay (RGA)

The luc2P/NFAT-RE/Hygro sequence was obtained from the pGL4.30-luc2P/NFAT-RE/Hygro vector (Promega). Lentivirus containing this sequence was generated by GenePharma with an integration titer of 1 × 10^8^ TU/ml. Jurkat cells (ATCC) were transduced with this lentivirus (MOI = 20) at 3 × 10^5^ cells/ml with RPMI 1640 (Gibco) containing 10% FBS and 8 *μ*g/ml polybrene. Twenty-four hours after transduction, the medium was removed and replaced with fresh medium containing 100 *μ*g/ml hygromycin, and cells were cultured for two weeks to generate stable cells. The cell line was maintained in medium containing 100 *μ*g/ml hygromycin.

For the RGA assay, 2.5 × 10^4^ cells were plated into 96-well plates (100 *μ*l/well) in medium containing other reagents. After 24 h, the medium was removed, and 1.5 × 10^5^ engineered Jurkat cells were added to wells with 100 *μ*l of medium containing the OKT3 CD3 agonist antibody (PeproTech). Cells were incubated for 8 h at 37°C and 5% CO_2_. Subsequently, 100 *μ*l of Promega Bio-GloTM Luciferase Assay was added to each well. After shaking the plate, a plate reader was used to measure the relative luciferase units (RLUs).

### 2.7. CD3+ T Cell-Induced Cytotoxicity Assay

Fresh human peripheral blood mononuclear cells (PBMCs) were purchased from Sailybio (Shanghai, China), and CD3+ T cells were separated by CD3 microbeads (Miltenyi Biotec). Tumor cells (2 × 10^4^; target) were plated in triplicate into 96-well plates and cocultured with CD3+ T cells (Effector) at a ratio of 2 : 1 (effector : target (E : T)) in 1640/RPMI medium containing 10% FBS. After incubation for 8 h, lactate dehydrogenase (LDH) from damaged cells was measured by a LDH Cytotoxicity Assay Kit (Beyotime). The absorbance was read with a microplate reader (Biotech Instruments Inc. USA) at 490 nm. The percent of cytotoxicity was calculated according the following formula: cytotoxicity% = (samples − E only blank)/(T only blank) × 100.

### 2.8. IFN-*γ* and IL-2 Analysis

Supernatants were collected from the cytotoxicity assay, and the concentrations of interleukin-2 (IL-2) and interferon-*γ* (IFN-*γ*) were measured using ELISA kits (Abcam).

### 2.9. Dual-Luciferase Reporter Assay

Luciferase activity assays were performed as previously described [14]. Briefly, HEK-293T cells were plated into a 96-well plate at 1 × 10^5^ cells/well and incubated for 24 h at 37°C and 5% CO_2_. Cells were then cotransfected with 200 ng of luciferase reporter vectors (Promega) containing target sequences and miRNAs. After 48 h, cells were lysed, and the luciferase activity was measured with the Dual-Luciferase Reporter Assay System (Promega).

### 2.10. RNA Immunoprecipitation (RIP)

The RIP assay was performed using the Magna RIP RNA-Binding Protein Immunoprecipitation Kit (Millipore). The lysate from 3 × 10^7^ cells was prepared with 100 *μ*l of RIP lysis buffer. After centrifugation, the supernatant was collected and incubated with RIP wash buffer, and the subsequent procedures were performed according to the manufacturer's instructions. The following antibodies were used for the RIP assay: anti-AGO2 (1 : 20) and control IgG (1 : 20) (Abcam). qPCR was used to detect enriched RNA.

### 2.11. RNA Pull-Down

Briefly, 50 nM biotinylated hsa-miR-582-5p (GenePharma) was transfected into Hep3B cells for 24 h by Lipofectamine 3000 reagents (Thermo Fisher Scientific). Then 1 × 10^7^ cells were harvested and lysed in lysis buffer, and the cell lysates were incubated with washed streptavidin magnetic beads (Life Technologies) at room temperature for 2 h. Beads were washed briefly three times, and TRIzol reagent (Invitrogen) was used to extract RNA. The coprecipitated RNA was analyzed by qPCR.

### 2.12. Flow Cytometry

To detect the GFP signal, cells were analyzed using a FACSCano II instrument (BD Biosciences) after washing once with PBS. Apoptosis assays were performed using an Annexin V-FITC/PI apoptosis detection kit (Beyotime). Briefly, 1 × 10^5^ cells were incubated with 200 *μ*l of buffer containing 5 *μ*l of Annexin V-FITC and 10 *μ*l of PI for 20 min at RT. Cells were then immediately placed on ice. Cells were washed once with PBS and analyzed on a FACSCano II instrument (BD Biosciences). The data were analyzed by FlowJo.

### 2.13. Cell Proliferation Assay

Cell proliferation was determined using a Cell Counting Kit-8 (CCK-8, Beyotime) assay according to the manufacturer's instructions. Briefly, cells were plated into 96-well plates at 5 × 10^3^ cells per well and cultured for 24 h. siRNAs or miRNAs were then transiently transfected into cells and incubated for 24, 48, 72, 96, and 120 h. At each time point, 10 *μ*l of CCK8 solution was added to each well and incubated for 2 h at 37°C. The absorbance was measured at 450 nm by a microplate reader. All experiments were repeated at least three times.

### 2.14. 5-Ethynyl-2-Deoxyuridine (EdU) Assay

Cells were labeled with 100 *μ*M EdU-labeling medium (RiboBio, China) at 1 × 10^4^ cells/well in 96-well plates. After 12 h or 24 h, cells were then treated with 4% paraformaldehyde and 0.5% Triton X-100 for 30 min followed by incubation with an anti-EdU working solution for 1 h. Nuclei were stained with DAPI. Images were acquired using a fluorescence microscope.

### 2.15. Migration and Invasion Assay

For the migration assay, 2 × 10^5^ cells in 300 *μ*l of serum-free medium were plated into the upper Transwell chamber, and 0.5 ml of medium containing 30% FBS was added to the lower chamber. After 48 h, cells on the underside of the membrane were fixed and stained with crystal violet solution (Beyotime) for 2 h at 37°C. Images were acquired using a microscope.

For the invasion assay, the steps were similar to those of the migration assay, except for one step. Before cell seeding, Matrigel (Corning) was coated onto the membrane on the upper Transwell chamber for 1 h at 37°C.

### 2.16. Xenograft Model

Six-week-old NCG mice were obtained from GemPharmatech (Jiangsu, China). Mice were randomly divided into 4 groups (*n* = 6) after adopting the new environment for 2 weeks. Cells (1 × 10^7^ cells/mouse) were subcutaneously injected into the back region of the mouse. At the same time, 100 *μ*l of PBS with or without PBMCs (1 × 10^7^) was injected into the mouse via the tail vein. Two weeks after xenografting, in which the tumor volume reached 100-200 mm^3^, cholesterol-coupled siRNAs (GenePharma) were intratumorally injected into the tumor every 4 days for 20 days. The tumor volume was measured every 4 days after the first siRNA injection using a Vernier caliper, and the tumor volume was calculated with the following formula: volume = length × width^2^/2. Tumor weight was determined at 30 days after the first siRNA injection.

All methods were performed following the guidelines and regulations of the Hospital of Chengdu University of Traditional Chinese Medicine Animal Care and Use Committee and the National Institutes of Health.

### 2.17. Statistical Analysis

Data were analyzed with the Student's *t* test using Prism (version 5; GraphPad Software). Comparisons of multiple groups were performed using one-way ANOVA followed by Tukey's multiple comparisons test. All data were assessed for normality of distribution using the Shapiro–Wilk test. The results are expressed as the mean ± SD (ns: *P* > 0.05; ^∗^: *P* < 0.05; ^∗∗^: *P* < 0.01; and ^∗∗∗^: *P* < 0.005. Values were considered statistically significant if *P* < 0.05).

## 3. Results

### 3.1. TUG1 Functions as a Mediator by Regulating Siglec-15 in HCC Cells

**Figure 1:**
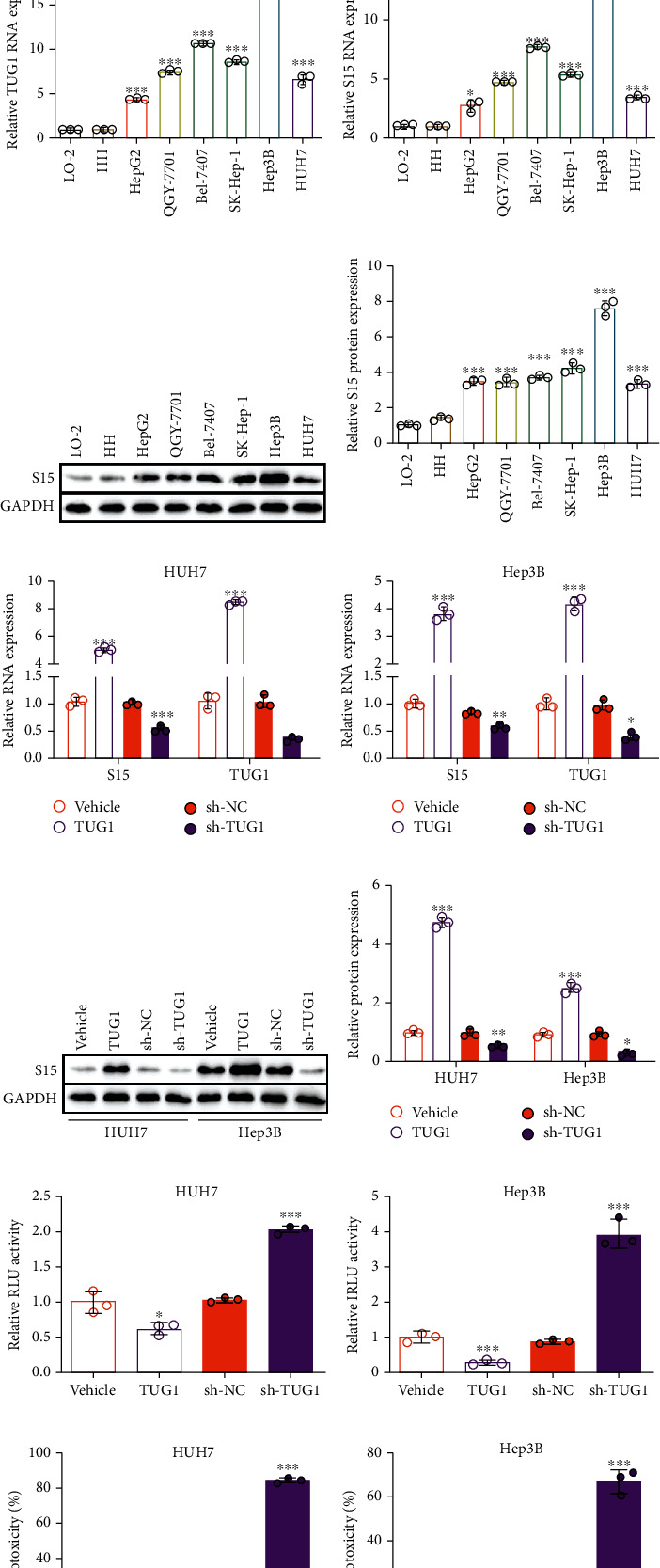
TUG1 is elevated in HCC cells and controls the Siglec-15 expression. (a) Schematic diagram of lncRNAs screen by bioinformation. (b) TUG1 expression in HCC patients from TCGA database. (c) Coexpression relationship between TUG1 and Siglec-15 in clinical data from TCGA database. (d) TUG1 expression, (e) Siglec-15 mRNA expression, and (f, g) protein expression in different HCC cell lines (HepG2, QGY-7701, Bel-7407, SK-Hep-1, Hep3B, and HUH7), human normal hepatic cell line LO-2, and primary human hepatocytes HH. (h, i) Siglec-15 mRNA expression and (j, k) protein expression in HUH7 and Hep3B cells treated with TUG1 overexpression or knockdown. (l, m) Relative luciferase activity of Jurkat-RGA in HUH7 and Hep3B cells treated with TUG1 overexpression or knockdown. (n, o) T cell-induced cytotoxicity cocultured with HUH7 or Hep3B cells treated with TUG1 overexpression or knockdown. (p, q) IFN-*γ* and (r, s) IL-2 secreted by T cells from (n, o). The data are presented as the means ± SD, *n* = 3 experiments in (d–s). ^∗^*P* < 0.05, ^∗∗^*P* < 0.01, ^∗∗∗^*P* < 0.005. S15: Siglec-15.

Siglec-15 has been identified as a new immune checkpoint and is considered a next-generation immunooncology agent [6, 7]. Considering the importance of Siglec-15 in cancer, we explored its regulatory role in HCC, especially in lncRNA-controlled pathways. Therefore, we screened potential lncRNAs through bioinformatics methods following the workflow shown in Figure 1(a). From TCGA database, we found 2 lncRNAs, TUG1 and MCM3AP-AS1, that affected the survival curve in HCC (TUG1, *P* = 0.024; and MCM3AP-AS1, *P* = 0.0098). Compared to the 32 search results of MCM3AP-AS1 plus Cancer in PubMed, 262 search results of TUG1 plus Cancer showed a clearer regulatory mechanism in tumor development. Thus, we focused on TUG1 as our target. The clinical expression and correlation with Siglec-15 of TUG1 are shown in Figures 1(b) and 1(c) (data from TCGA and analyzed by Pan-Cancer program in the Starbase web site).

We next analyzed the TUG1 expression in six HCC cell lines (HepG2, QGY-7701, Bel-7407, SK-Hep-1, Hep3B, and HUH7), the LO-2 human normal hepatic cell line, and the HH primary human hepatocyte line. A significantly higher level of TUG1 was present in the HCC cell lines compared to the HH and LO-2 cells with the highest expression in Hep3B cells (Figure 1(d)). Consistently, a similar tendency was found for Siglec-15 expression in these cells at both the mRNA and protein levels (Figures 1(e)–1(g)). A positive correlation between TUG1 and Siglec-15 expression was identified as shown in Figures 1(d) and 1(e). We constructed stable cell lines with TUG1 overexpression or TUG1 knockdown using HUH7 (low endogenous TUG1 expression) and Hep3B (high endogenous TUG1 expression). In both Hep3B and HUH7 cells, high TUG1 expression resulted in the upregulation of Siglec-15 expression, and shRNA-induced TUG1 knockdown led to Siglec-15 downregulation, including both the mRNA and protein levels (Figures 1(h)–1(k)).

Moreover, we performed a series of assays to evaluate the anti-immune response in engineered Hep3B and HUH7 cells. The Jurkat-based RGA assay is a method to estimate the signal strength of T cell activity. Jurkat is a T cell line that can be activated by tumor cells, but it lacks a cytotoxic effect. Thus, the NF-*κ*B-controlled luciferase expression in Jurkat cells reflects the signal strength excited by tumor cells, stimulating the T cell response. The RGA results demonstrated higher luciferase activity in cells overexpressing TUG1 but lower luciferase activity in TUG1-deficient cells, indicating that TUG1-induced Siglec-15 regulation affects the signal strength in the T cell response (Figures 1(l) and 1(m)). We also performed T cell-induced cytotoxicity in engineered Hep3B and HUH7 cells to further explore the role of TUG1 in the immunoreaction. We found elevated cytotoxicity in TUG1 knockdown cells and an adverse effect in TUG1-overexpressing cells (Figures 1(n) and 1(o)). Similar results were observed for the levels of IFN-*γ* and IL-2, cytokines secreted by T cells to execute cytotoxicity (Figures 1(p)–1(s)).

These results illustrated that TUG1 functions as a negative regulator in the T cell-induced immune response by regulating Silgec-15 expression.

### 3.2. hsa-miR-582-5p Is Responsible for the Siglec-15 Regulation Induced by TUG1

**Figure 2:**
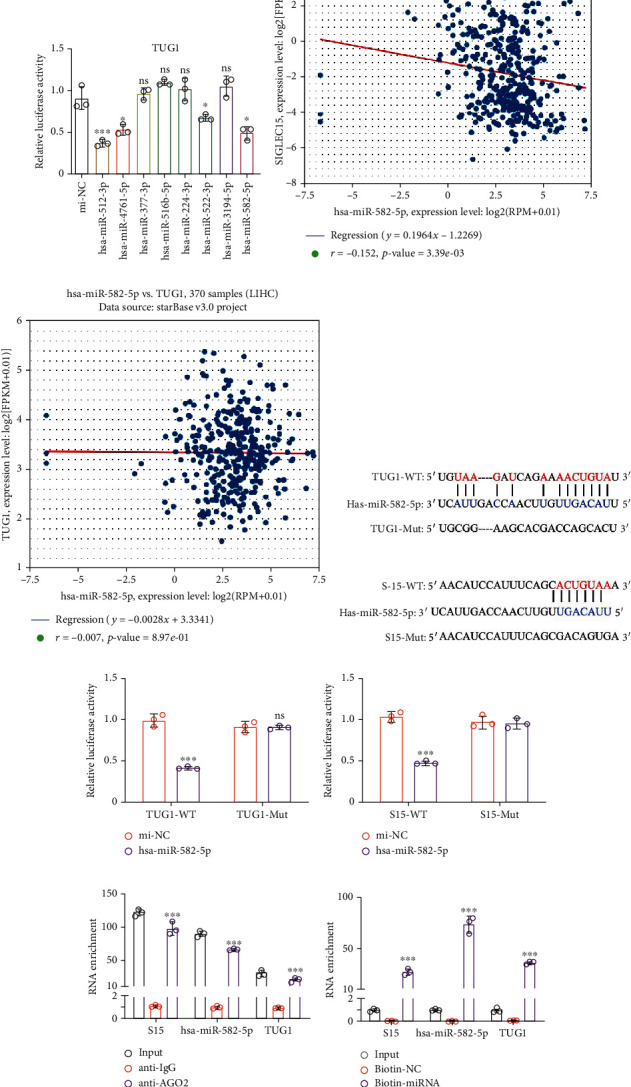
hsa-miR-582-5p directly binds to TUG1 and 3′-UTR of Siglec-15. (a) The intersection of predicted miRNAs targeting TUG1 and Siglec-15. Relative luciferase activity of dual-luciferase assay in miRNAs screen targeting (b) Siglec-15 and (c) TUG1. The coexpression relationship between hsa-miR-582-5p and (d) Siglec-15 or (e) TUG1 in clinical data from TCGA database. (f) The predicted binding sequence between hsa-miR-582-5p and TUG1 or Siglec-15. Relative luciferase activity of dual-luciferase assay in hsa-miR-582-5p targeting (g) Siglec-15 and (h) TUG1. (i) RIP assay in Hep3B cells coprecipitated with AGO2 protein. (j) RNA pull-down assay in Hep3B cells coprecipitated with biotin-labeled hsa-miR-582-5p. (k) Relative luciferase activity of dual-luciferase assay in hsa-miR-582-5p targeting Siglec-15 with TUG1 regulation. The data are presented as the means ± SD, *n* = 3 replicates in (b, c, and g–k). ^∗^*P* < 0.05, ^∗∗^*P* < 0.01, ^∗∗∗^*P* < 0.005. S15: Siglec-15.

One of the common mechanisms by which lncRNAs regulate downstream genes is through a lncRNA-miRNA-mRNA ceRNA network. Thus, we hypothesized that there was a miRNA between TUG1 and Siglec-15. After intersecting the predicted miRNAs targeting TUG1 and Siglec-15 by the TargetScan and starBase databases, we found that a total of 8 miRNAs had the potential to target both of them simultaneously (Figure 2(a)).

We subsequently screened those miRNAs using a luciferase reporter assay. The results showed that hsa-miR-224-3p and hsa-miR-582-5p could target the 3′-URT of Siglec-15, while hsa-miR-512-3p, hsa-miR-4761-5p, hsa-miR-522-3p, and hsa-miR-582-5p could target TUG1 (Figures 2(b) and 2(c)). hsa-miR-582-5p was the only miRNA capable of targeting TUG1 and Siglec-15 simultaneously. We also found a negative correlation between hsa-miR-582-5p and Siglec-15 expression in TCGA database, but hsa-miR-582-5p showed no correlation with TUG1, indicating sponge activity between TUG1 and hsa-miR-582-5p (Figures 2(d) and 2(e)). The predicted binding format is shown in Figure 2(f). We also performed a luciferase assay using mutant groups (Mut) to confirm the interaction. The luciferase activity of TUG1-WT or Siglec-15-WT was inhibited in the presence of hsa-miR-582-5p with no difference in the Mut groups, suggesting that hsa-miR-582-5p targets TUG1 and the 3′-UTR of Siglec-15 simultaneously (Figures 2(g) and 2(h)).

Furthermore, the RIP assay showed that Siglec-15 mRNA, TUG1, and hsa-miR-582-5p were enriched and coprecipitated with AGO2 protein (Figure 2(i)), indicating that they are involved in the AGO2 complex. We then used an RNA pull-down assay to investigate the interaction among Siglec-15 mRNA, TUG1, and hsa-miR-582-5p. In Hep3B cells, a biotin-labeled hsa-miR-582-5p pulled down TUG1 and also pulled down Siglec-15 mRNA (Figure 2(j)). These findings demonstrated that hsa-miR-582-5p directly and simultaneously binds to TUG1 and the 3′-UTR of Siglec-15. Moreover, we also found that the hsa-miR-582-5p-induced reduction of luciferase activity of the wild-type 3′-UTR in the Siglec-15 groups was transiently reversed by TUG1 cotransfection (Figure 2(k)) without any changes in the mutant groups. Together, these results indicated that TUG1 may sponge hsa-miR-582-5p to regulate Siglec-15 expression.

### 3.3. hsa-miR-582-5p Regulates the T Cell-Induced Immune Response of HCC Cells

**Figure 3:**
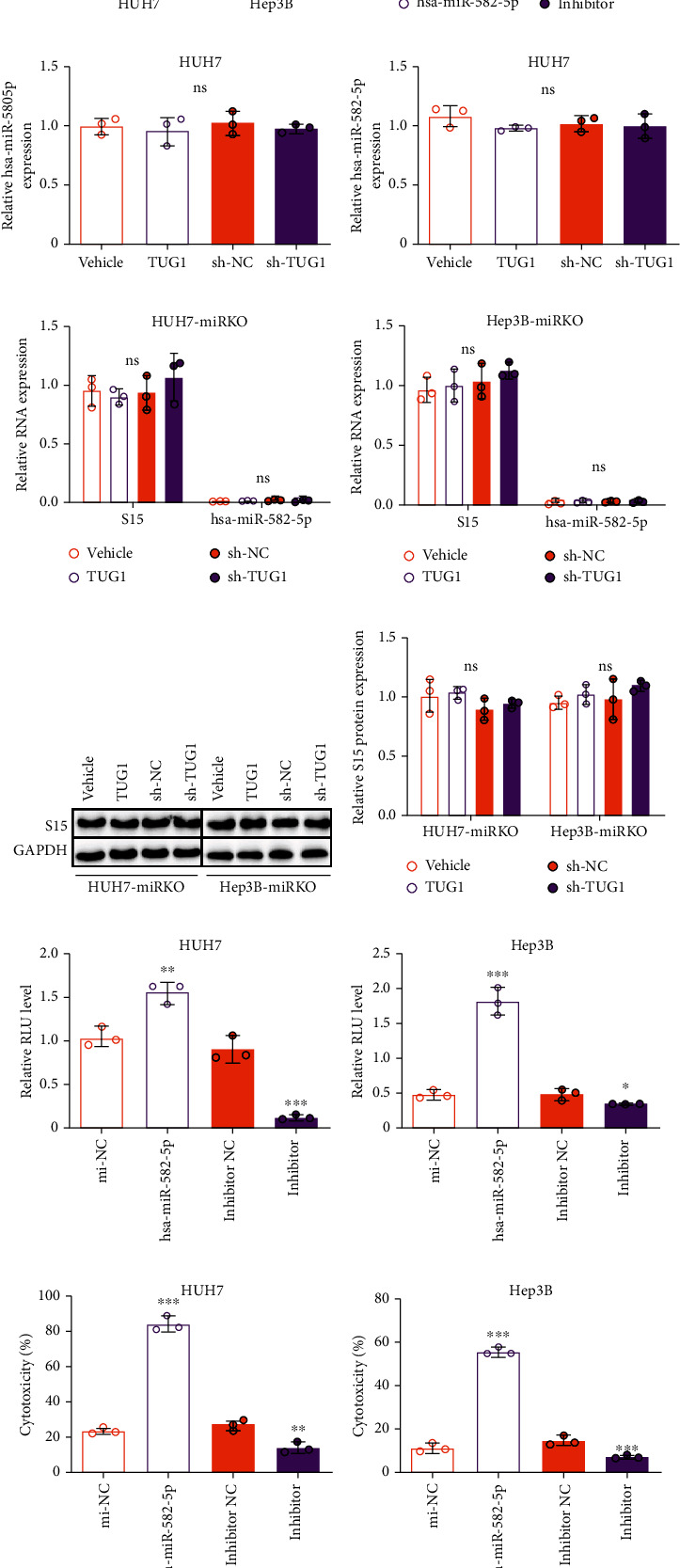
hsa-miR-582-5p negatively regulates Siglec-15 level and its induced immunosuppression. (a) hsa-miR-582-5p expression in HCC patients and normal person from GEO analysis (GPL8179). (b) hsa-miR-582-5p in different HCC cell lines (HepG2, QGY-7701, Bel-7407, SK-Hep-1, Hep3B, and HUH7), human normal hepatic cell line LO-2, and primary human hepatocytes HH. (c, f) TUG1 expression, Siglec-15 mRNA expression, and protein expression in HUH7 and Hep3B cells treated with hsa-miR-582-5p mimics or inhibitor. hsa-miR-582-5p expression in (g) HUH7 and (h) Hep3B cells treated TUG1 overexpression or knockdown. (i, j) hsa-miR-582-5p expression, (i, j) Siglec-15 mRNA, and (k, l) protein expression in HUH7-miRKO and Hep3B-miRKO cells treated TUG1 overexpression or knockdown. (m, n) Relative luciferase activity of Jurkat-RGA in HUH7 and Hep3B cells treated with hsa-miR-582-5p mimics or inhibitor. (o, p) T cell-induced cytotoxicity cocultured with HUH7 and Hep3B cells treated with hsa-miR-582-5p mimics or inhibitor. (q, r) IFN-*γ* and (s, t) IL-2 secreted by T cells from (o, p). The data are presented as the means ± SD, *n* = 3 replicates in (b–t). ^∗^*P* < 0.05, ^∗∗^*P* < 0.01, ^∗∗∗^*P* < 0.005. S15: Siglec-15.

Interestingly, a GEO analysis (GPL8179) has shown that hsa-miR-582-5p has a 0.53-fold reduction in HCC patients compared to healthy individuals (*P* = 0.028) (Figure 3(a)) [15]. We also measured the expression of hsa-miR-582-5p in 6 HCC cell lines, LO-2 cells, and HH cells. We found a general decrease in hsa-miR-582-5p expression in the HCC cell lines compared to LO-2 and HH cells (Figure 3(b)). Next, we explored the effects of hsa-miR-582-5p on Siglec-15 and TUG1 expression. qPCR and western blot analyses showed that transfection with hsa-miR-582-5p inhibited Siglec-15 abundance, while the inhibitor of hsa-miR-582-5p increased Siglec-15 expression, indicating that hsa-miR-582-5p negatively regulates Siglec-15 expression (Figures 3(c)–3(f)). However, TUG1 expression displayed no significant changes with the variable hsa-miR-582-5p level (Figures 3(c) and 3(d)). We found that the artificially controlled TUG1 level in Hep3B and HUH7 cells failed to affect hsa-miR-582-5p expression (Figures 3(g) and 3(h)). The noninterference performances between TUG1 and hsa-miR-582-5p were in accordance with the ceRNA mechanism.

To further confirm the necessity of hsa-miR-582-5p in TUG1-induced Siglec-15 regulation, we used hsa-miR-582-5p knockout HUH7 and Hep3B cell lines (HUH7-miRKO and Hep3B-miRKO, respectively). Notably, the expression of Siglec-15 remained unchanged with the variable TUG1 level in hsa-miR-582-5p knockout cell lines at either the mRNA or protein level, indicating that Siglec-15 expression is not controlled by TUG1 when hsa-miR-582-5p is knocked out (Figures 3(i)–3(l)). These results suggested that hsa-miR-582-5p is necessary for TUG1-regulated Siglec-15 expression.

In addition, we evaluated the role of hsa-miR-582-5p in the immune response in HCC cells. hsa-miR-582-5p overexpression increased the luciferase activity in the RGA assay, but the inhibitor of hsa-miR-582-5p reduced the luciferase activity (Figures 3(m) and 3(n)). Similarly, higher levels of cytotoxicity as well as IFN-*γ* and IL-2 concentrations were found in HCC cells overexpressing hsa-miR-582-5p, and the opposite effect was found when treated with the inhibitor of hsa-miR-582-5p (Figures 3(o)–3(t)). These findings demonstrated that hsa-miR-582-5p impacts the T cell-induced immune response in HCC cells by regulating Siglec-15 expression.

### 3.4. siRNAs Screen for Developing RNAi Drugs

Our initial aim was to identify a target for siRNA drugs in HCC therapy by inhibiting Siglec-15 abundance. Except for the function of TUG1 in Siglec-15 regulation identified in our study, other studies have demonstrated that TUG1 is an oncogene that promotes cancer development and increases tumor malignancy, including tumor cell survival, proliferation, migration, and invasion, in cancers such as HCC [10–13]. Thus, TUG1 has a dual role in cancer development as it promotes the anti-immune response and oncogenicity, indicating that it is an ideal target for siRNA drug development. Once a siRNA knocks down TUG1 in HCC, different mechanisms will be working to eliminate the tumor by suppressing its progression and improving the immune response. Therefore, we began to develop a si-TUG1 sequence with high knockdown efficiency.

**Figure 4:**
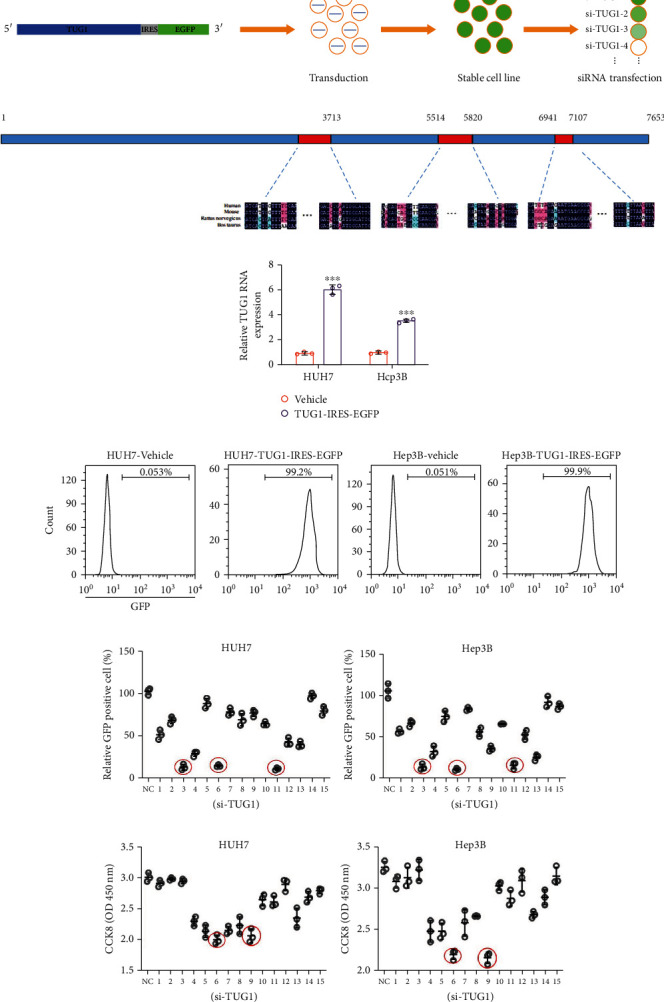
The screen of siRNAs targeting TUG1. (a) Schematic diagram of the siRNA screen method and cell line construction. (b) Schematic diagram of the TUG1 conserved region. (c) TUG1 expression in HUH7-TUG1-IRES-EGFP and Hep3B-TUG1-IRES-EGFP cells. (d) Flow cytometry results of EGFP positive rate in HUH7-TUG1-IRES-EGFP and Hep3B-TUG1-IRES-EGFP cells. The screen of siRNAs targeting TUG1 in (e) HUH7-TUG1-IRES-EGFP and (f) Hep3B-TUG1-IRES-EGFP (F) cells by flow cytometry. The screen of siRNAs targeting TUG1 in (g) HUH7 and (h) Hep3B cells by CCK8. The data are presented as the means ± SD, *n* = 3 replicates in (c and e–h). ^∗^*P* < 0.05, ^∗∗^*P* < 0.01, ^∗∗∗^*P* < 0.005. S15: Siglec-15.

First, we designed a high-throughput method using HUH7 and Hep3B cells stably expressing TUG1-IRES-EGFP genes using the EGFP signaling to replace the TUG1 abundance. Once siRNA candidates were capable of TUG1 knockdown, EGFP expression was inhibited, allowing siRNAs to be screened by flow cytometry (Figure 4(a)). Second, the target region for siRNAs was predicted to be the conserved region in the TUG1 sequence, thereby avoiding efficacy loss due to mutation. We aligned the TUG1 sequences in humans, mice, rats, and cattle from the National Center of Biotechnology Information (NCBI). The regions of 3453-3713 bp, 5514-5820 bp, and 6941-7107 bp in the human TUG1 sequence were conserved regions with high homology among different species (Figure 4(b)). Fifteen siRNAs were designed according to the conserved regions by siDirect (http://sidirect2.rnai.jp/) (Table 1). The cell lines stably expressing TUG1-IRES-EGFP were confirmed by qPCR (measuring TUG1 expression) and flow cytometry (measuring EGFP expression) (Figures 4(c) and 4(d)). After the siRNA screen, we found that si-TUG1-3, si-TUG1-6, and si-TUG1-11 displayed the best level of TUG1 inhibition in both HUH7 and Hep3B cells (Figures 4(e) and 4(f)).

Because many studies have indicated that TUG1 inhibits tumor cell proliferation in HCC [10, 16], we used a CCK-8 assay to screen the performance of the 15 siRNAs 72 h after transfecting them into wild-type HUH7 and Hep3B cells. The results indicated that si-TUG1-6 and si-TUG1-9 inhibited cell proliferation better than the other siRNAs (Figures 4(g) and 4(h)). Because si-TUG1-6 had the strongest effect on TUG1 downregulation and cell proliferation inhibition in HCC cells, we used it for subsequent experiments.

### 3.5. si-TUG1-6 Suppresses Tumor Development by Reducing Tumor Cell Proliferation and Increasing the Immune Response

**Figure 5:**
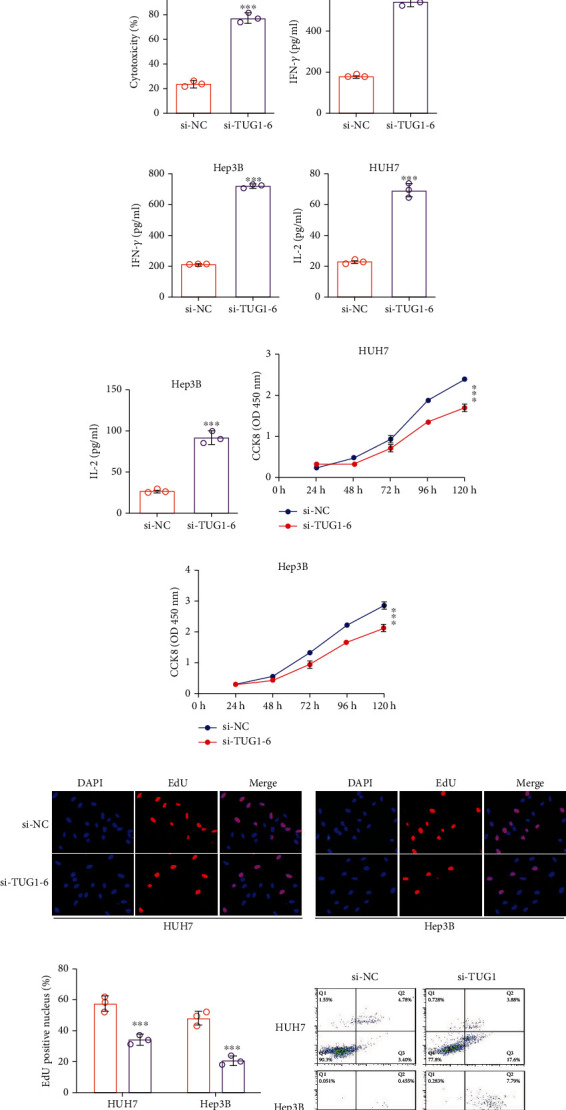
The antitumor activity of si-TUG1-6 in vitro. (a, b) The immunofluorescence results of HUH7-TUG1-IRES-EGFP and Hep3B-TUG1-IRES-EGFP cells treated with si-TUG1-6. (c) TUG1 expression, (d) Siglec-15 mRNA, and (e, f) protein expression in HUH7 and Hep3B cells treated si-TUG1-6. (g, h) Relative luciferase activity of Jurkat-RGA in HUH7 and Hep3B cells treated with si-TUG1-6. (i, j) T cell-induced cytotoxicity cocultured with HUH7 and Hep3B cells treated with si-TUG1-6. (k, l) IFN-*γ* and (m, n) IL-2 secreted by T cells from (i, j). (o, p) Cell proliferation in HUH7 and Hep3B cells treated with si-TUG1-6 analyzed by CCK8. (q, r) Cell proliferation in HUH7 and Hep3B cells treated with si-TUG1-6 analyzed by EdU assay. (s, t) Cell apoptosis in HUH7 and Hep3B cells treated with si-TUG1-6 analyzed by Annexin V/PI. (u, v) Apoptosis-relevant proteins level in HUH7 and Hep3B cells treated si-TUG1-6. (w–z) (q, r) Migration and (s, t) invasion of HUH7 and Hep3B cells treated with si-TUG1-6. The data are presented as the means ± SD, *n* = 3 replicates in (a–v), *n* = 10 samples in (w–z). ^∗^*P* < 0.05, ^∗∗^*P* < 0.01, ^∗∗∗^*P* < 0.005. S15: Siglec-15.

To verify the function of si-TUG1-6, we first transfected it into HCC cells overexpressing TUG1-IRES-EGFP. The immunofluorescence results showed a remarkable reduction in EGFP expression (Figures 5(a) and 5(b)). Next, we knocked down TUG1 expression in wild-type HUH7 and Hep3B cells with si-TUG1-6, resulting in a nearly 80% reduction. Moreover, the Siglec-15 level was reduced by 70-80% in cells treated with si-TUG1-6 (Figures 5(d)–5(f)). These results suggested that si-TUG1-6 is highly efficient in suppressing TUG1 levels in HCC cells.

Subsequently, we evaluated the effects of si-TUG1-6 on the immune response in HUH7 and Hep3B cells. si-TUG1-6 caused the luciferase activity in the Jurkat-RGA assay to significantly increase, which was consistent with the T cell-induced cytotoxicity and cytokine release (Figures 5(g)–5(n)).

In addition, we also evaluated the function of si-TUG1-6 in tumor oncogenicity in HCC cells. We found that si-TUG1-6 overexpression severely inhibited the proliferation of HUH7 and Hep3B cells (Figures 5(o)–5(r)). The apoptosis of HUH7 and Hep3B cells was also increased after treatment with si-TUG1-6 (Figures 5(s) and 5(t)), consistent with upregulated C-caspase3 and Bcl-2 protein levels as well as reduced caspase3 protein levels (Figures 5(u) and 5(v)). The migration and invasion abilities of HUH7 and Hep3B cells were also measured, and the results showed that si-TUG1-6 effectively repressed malignant tumor behavior (Figures 5(w)–5(z)).

Together, these findings suggested that si-TUG1-6 is an efficient antitumor inhibitor by suppressing tumor progression and improving the immune response via downregulating Siglec-15 in HCC cells in vitro.

### 3.6. si-TUG1-6 Exhibits a Synergistic Antitumor Effect in HCC In Vivo

**Figure 6:**
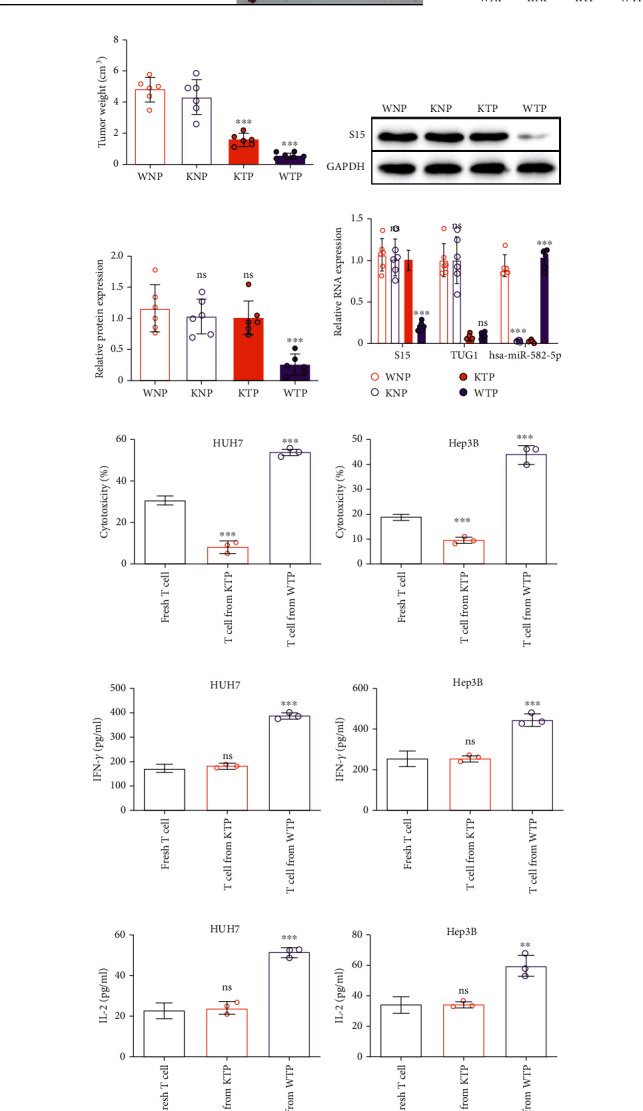
The antitumor activity of si-TUG1-6 in vivo. (a) The image of tumor growth the introduction of each group. WNP: NCG mice injected with wide-type Hep3B cells, si-NC, and human PBMC; KNP; NCG mice injected with Hep3B-miRKO cells, si-NC, and human PBMC; KTP: NCG mice injected with Hep3B-miRKO cells, si-TUG1-6, and human PBMC. WTP: NCG mice injected with wide-type Hep3B cells, si-TUG1-6, and human PBMC. (b) Tumor volume and (c) tumor wright in each group. (d, e) Siglec-15 protein expression in tumors from each group. (f) RNA expression of Siglec-15, hsa-miR-582-5p, and TUG1 in tumors from each group. (g, h) Cytotoxicity of T cells from mice in each groups and cocultured with HUH7 and Hep3B cells. (i, j) IFN-*γ* and (k, l) IL-2 secreted by T cells from (g, h). The data are presented as the means ± SD, *n* = 6 samples and 3 replicates in (b–f), *n* = 3 replicates in (g–k). ^∗^*P* < 0.05, ^∗∗^*P* < 0.01, ^∗∗∗^*P* < 0.005. S15: Siglec-15.

A xenograft model in NCG mice was used to evaluate the antitumor efficacy of si-TUG1-6 in HCC with the following groups (Figure 6(a)): WNP, mice were injected with wild-type Hep3B cells (WT, W), si-NC (N), and PBMCs (P); KNP, mice were injected with Hep3B-miRKO cells (miRKO, K), si-NC (N), and PBMCs (P); KTP, mice were injected with Hep3B-miRKO cells (miRKO, K), si-TUG1-6 (T), and PBMCs (P); WTP, mice were injected with wild-type Hep3B cells (WT, W), si-TUG1-6 (T), and PBMCs (P); WNN, a control group to reflect original tumor growth in PBMCs; and KNN, a control group to demonstrate the effect of hsa-miR-582-5p knockout on tumor growth in PBMCs. The KTP group was designed to eliminate the influence of si-TUG1-6 on Siglec-15 in tumor cells as T*Μ*G2 failed to disturb Siglec-15 expression with hsa-miR-582-5p knockout in vitro (Figures 3(i)–3(l)). Therefore, the change in tumor growth in the KTP group was only attributed to the tumor cell oncogenicity induced by si-TUG1-6 compared to the WNP and KNP groups. WTP was a group designed to reflect the synergetic effects on tumor growth induced by si-TUG1-6.

There were no significant differences between the WNN and KNN groups, which indicated that hsa-miR-582-5p knockout did not affect tumor growth in HCC (Figures 6(a)–6(c)). The results of the KTP group, which showed reduced tumor growth compared to the WNN and KNN groups, demonstrated that this reduction was due to the inhibition of tumor progression induced by si-TUG1-6 and that hsa-miR-582-5p knockout did not affect tumor growth (Figures 6(a)–6(c)). More importantly, the WTP group showed more suppression of tumor growth than the KTP group, indicating that the increased antitumor activity was attributed to si-TUG1-6-induced Siglec-15 downregulation (Figures 6(a)–6(c)). These results revealed that si-TUG1-6 displays a synergetic antitumor effect by inhibiting tumor progression and increasing immune activity.

In addition, we also measured the expression of Siglec-15 and TUG1 in tumors from different groups. The results showed no change in Siglec-15 levels in all groups, except the WTP group (Figures 6(d) and 6(e)), which showed decreased Siglec-15 expression (Figures 6(d)–6(f)). TUG1 expression was only reduced in tumors treated with si-TUG1-6 (Figure 6(f)). Moreover, hsa-miR-582-5p level was not expressed in the miRKO group (Figure 6(f)). These results support the conclusion drawn from Figures 6(a)–6(c).

To further analyze the influence of Siglec-15 levels on T cell cytotoxic activity, we isolated PBMCs from KTP and WTP mice at the endpoint. We then detected CD3+ T cell activity isolated from PBMCs through a T cell cytotoxicity assay. Compared to fresh CD3+ T cells, there was a reduction in CD3+ T cells and a higher cytotoxicity activity in CD3+ T cells from the WTP group (Figures 6(g) and 6(h)). Similar results were also found for cytokine release, and there was no difference between fresh CD3+ T cells and CD3+ T cells from the KTP group (Figures 6(i)–6(l)). These results suggested that CD3+ T cells from the KTP group may be exhausted due to Siglec-15 and other immune checkpoint molecules. In contrast, the good performance of CD3+ T cells from the WTP group in the immune response in vitro may be attributed to removing Siglec-15-induced restrictions on T cell activity.

Together, these in vivo results demonstrated that si-TUG1-6 displays antitumor activity in HCC with a synergetic effect by inhibiting tumor progression and increasing immune activity via repressing Siglec-15 expression.

## 4. Discussion

Antibodies have become one of the most effective pharmaceuticals for treating human diseases, including cancers. Among the antibody drugs for treating cancers, one type of drug targeting immune checkpoints displays promising therapeutic effects on many cancers, such as lung cancer or breast cancer [17]. Many immune checkpoint antigens are used in antibody drugs, such as PD1/PD-L1, LAG-3, and CTLA-4/CD80/CD86 [17]. These antibody drugs increase the immune response to cancers by blocking cancer-induced immunosuppression in the body after binding to immune checkpoint antigens. Thus, a substitute strategy may work to achieve the same purpose by knocking down immune checkpoint expression using siRNAs [18]. Lian et al. treated lung metastasis using an epithelial cell adhesion molecule- (EpCAM-) targeted cationic liposome (LPP-P4-Ep) containing si-CD47 and si-PD-L1; they found that lung metastasis was reduced in the lung metastasis model [19].

Siglec-15 was initially characterized by Dr. Takashi Angata in 2007 as a unique member that is selectively expressed on myeloid cells and osteoclasts (a bone-specific myeloid lineage) but generally absent in other immune cells and tissues [5, 7, 20]. In 2019, the Lieping Chen group stated that Siglec-15 is an immune checkpoint comparable to PD-L1 with high expression in macrophages and many cancers, including liver cancers [7, 21]. Thus, Siglec-15 is called a next-generation immunooncology agent and has received much attention, especially in antibody drug development. However, the regulatory mechanism of Siglec-15 in cancers remains unclear.

Many studies have shown that TUG1 plays a significant role in tumor progression. Zhang et al. found that downregulation of TUG1 inhibits cell proliferation and promotes apoptosis in osteosarcoma [11]. Shao et al. showed that knockdown of TUG1 suppresses cell proliferation and migration through the KLF4/miR-153-1 axis [13]. Notably, TUG1 has also been demonstrated to promote cell migration, invasion, and proliferation in HCC through different pathways, such as the miR-29c-3p/COL1A1 axis [10], miR-137/AKT2 axis [22], and miR-144/JAK2/STAT3 axis [16]. However, the understanding of TUG1 in immune response regulation remains unknown. We first demonstrated the regulatory role of TUG1 in the immune response by upregulating the Siglec-15 level in HCC cells, aiding in the establishment of immunosuppression. These findings suggested that TUG1 is a dual-function target for RNAi drug development.

Cancer treatments composed of immune checkpoint inhibitors and oncogene-targeted drugs display a better therapeutic effect in clinical conditions, such as lung cancer, breast cancer, and colon cancer [23]. This strategy includes the following two antitumor mechanisms: inhibiting tumor progression and blocking immunosuppression. However, two or more drugs are needed to achieve this purpose, resulting in a greater possibility of toxicity and side effects. Thus, a single molecule with double antitumor functions may be safer and more effective. Our study demonstrated that TUG1 is an appropriate target because its knockdown displayed dual antitumor functions. We also developed an efficient siRNA targeting TUG1 (si-TUG1-6), which exhibited antitumor activity in vitro and in vivo. Combined with the GalNAc-siRNA conjugate technique, which has high safety and high delivery efficiency into hepatocytes in vivo [24–26], our study suggests that TUG1 is an appropriate target to treat HCC through siRNA drugs.

In conclusion, TUG1 plays a regulatory role in Siglec-15 expression in HCC cells by targeting and sponging hsa-miR-582-5p. TUG1-induced Siglec-15 upregulation increases the immunosuppression between HCC cells and T cells. Moreover, siRNA downregulation of TUG1 expression shows antitumor activity in HCC cells by suppressing tumor progression, such as proliferation, migration, and invasion, as well as by enhancing the T cell-induced immune response both in vitro and in vivo. These findings indicated that TUG1 is a potential and applicable target for RNAi drug development in HCC treatment.

## Figures and Tables

**Table 1:** siRNA sequences.

No.	Target sequence 21nt target + 2nt overhang	RNA oligo sequences 21nt guide (5′→3′) 21nt passenger (5′→3′)
3453 bp-3713 bp		
si-TUG1-1	GGCCTGAATCCTGCTACAACTAT	AGUUGUAGCAGGAUUCAGGCC
		CCUGAAUCCUGCUACAACUAU
si-TUG1-2	TGCTACAACTATCTTCCTTTACC	UAAAGGAAGAUAGUUGUAGCA
		CUACAACUAUCUUCCUUUACC
si-TUG1-3	TACAACTATCTTCCTTTACCACC	UGGUAAAGGAAGAUAGUUGUA
		CAACUAUCUUCCUUUACCACC
si-TUG1-4	TTCCTTACAACACCTTGAACTCT	AGUUCAAGGUGUUGUAAGGAA
		CCUUACAACACCUUGAACUCU

5514 bp-5820 bp		
si-TUG1-5	GCCTTGACTTGCTTGTAAGATGA	AUCUUACAAGCAAGUCAAGGC
		CUUGACUUGCUUGUAAGAUGA
si-TUG1-6	ACGACTTGATTACCAAAGAAAGT	UUUCUUUGGUAAUCAAGUCGU
		GACUUGAUUACCAAAGAAAGU
si-TUG1-7	GACTTGATTACCAAAGAAAGTAG	ACUUUCUUUGGUAAUCAAGUC
		CUUGAUUACCAAAGAAAGUAG
si-TUG1-8	TAGCATAGACTCCTAAACAGAAC	UCUGUUUAGGAGUCUAUGCUA
		GCAUAGACUCCUAAACAGAAC
si-TUG1-9	AGCATAGACTCCTAAACAGAACC	UUCUGUUUAGGAGUCUAUGCU
		CAUAGACUCCUAAACAGAACC
si-TUG1-10	CTGTAAGATCAGAAAACTGTATC	UACAGUUUUCUGAUCUUACAG
		GUAAGAUCAGAAAACUGUAUC

6941 bp-7107 bp		
si-TUG1-11	CTGGACTTTTCAGTTATGTGAAC	UCACAUAACUGAAAAGUCCAG
		GGACUUUUCAGUUAUGUGAAC
si-TUG1-12	TGGACTTTTCAGTTATGTGAACC	UUCACAUAACUGAAAAGUCCA
		GACUUUUCAGUUAUGUGAACC
si-TUG1-13	TTCAGTTATGTGAACCAATAAAT	UUAUUGGUUCACAUAACUGAA
		CAGUUAUGUGAACCAAUAAAU
si-TUG1-14	CAGTTATGTGAACCAATAAATAC	AUUUAUUGGUUCACAUAACUG
		GUUAUGUGAACCAAUAAAUAC
si-TUG1-15	AACCAATAAATACCCTTTTTTGC	AAAAAAGGGUAUUUAUUGGUU
		CCAAUAAAUACCCUUUUUUGC

## Data Availability

The data used to support the findings of this study are available from the corresponding authors upon reasonable request.
